# Suicidal ideation among mental health patients at hospital discharge: prevalence and risk factors

**DOI:** 10.1186/s12888-025-06547-3

**Published:** 2025-02-11

**Authors:** Wanying Mao, Reham Shalaby, Ernest Owusu, Hossam Eldin Elgendy, Belinda Agyapong, Ejemai Eboreime, Peter H. Silverstone, Pierre Chue, Xin-Min Li, Wesley Vuong, Arto Ohinmaa, Valerie Taylor, Andrew J. Greenshaw, Vincent Israel Opoku Agyapong

**Affiliations:** 1 Department of Psychiatry, University of Alberta, Edmonton, Canada; 2 Department of Psychiatry, Faculty of Medicine, Dalhousie University, 5909 Veterans Memorial Lane, 8th Floor Abbie J. Lane Memorial Building QEII Health Sciences Centre, Halifax, NS B3H 2E2 Canada; 3 Connect Care Clinical Operations Informatics Office, Alberta Health Services, Edmonton Zone, AB Canada; 4 School of Public Health, University of Alberta, Edmonton, AB Canada; 5 Department of Psychiatry, Cumming School of Medicine, University of Calgary, Calgary, AB Canada

**Keywords:** Suicide ideation, Psychiatry discharge, Mental health, Prevalence, Risk factors

## Abstract

**Background:**

Evidence indicates that suicide risk is much higher for psychiatric patients in the weeks immediately following discharge from the hospital. It is, therefore, crucial to evaluate suicide risk accurately at discharge to provide supportive and lifesaving interventions as appropriate. *Aim*: In this study, the prevalence and risk factors for suicide ideations were examined among patients ready to be discharged from psychiatric units in Alberta province, Canada.

**Methods:**

Researchers conducted face-to-face meetings with potential participants to determine if they were interested in participating. Eligible individuals in this epidemiological cross-sectional study used an online quantitative survey to assess suicide ideations using the appropriate question contained in the Patient Health Questionnaire (PHQ-9) scale. Information was also gathered regarding patient demographics, clinical information, and responses to the Generalized Anxiety Disorder (GAD-7), and World Health Organization Well-Being Index (WHO-5) questionnaires.

**Results:**

We recruited 1,004 patients from an initial pool of 1,437 patients. We found that the prevalence of suicidal ideation among patients about to be discharged was 48.9%, i.e., nearly half of all patients had active suicidal thinking prior to discharge. We found that factors that were most significantly associated with this were age, ethnicity, employment status, primary mental health diagnoses, anxiety, and poor well-being at baseline.

**Conclusion:**

Here, in a large cohort of psychiatric patients in Alberta, Canada, we found that nearly half of patients being discharged from an acute psychiatric unit reported suicidal ideation. Given the increased short-term risk to this group, there is an urgent need for additional research on the underlying reasons and reliable predictors of suicidal ideation in these patients. Additionally, appropriate interventions and supportive services must be provided both prior and after discharge to mitigate this substantial risk.

## Introduction

Suicidal ideations, often referred to as suicidal thoughts or ideas, encompass a range of contemplations, wishes, and preoccupations surrounding death and suicide [1]. The DSM-5 defines it as “thoughts about self-harm, with deliberate consideration or planning of possible techniques of causing one’s own death“ [2]. Generally, it is an expression of dissatisfaction with one’s existence or quality of life, with the intent of not being alive [3]. Based on a research published in 2019, it is estimated that 2.5% of Canadians had suicidal thoughts within the previous year, and around 11.8% of the population has expressed death wishes at some point in their lives [4]. There were approximately 3.1% of respondents who had attempted suicide in their lifetime, and 4.0% who had planned to commit suicide [5]. According to a Statistics Canada report, suicide impacts Canadians of all ages and backgrounds [6]. There are approximately 4,500 suicides in Canada every year, which is equivalent to 12 suicides per day. Every day, more than 200 people in Canada attempt suicide [6].

In the immediate aftermath of being discharged from a psychiatric hospital, suicide rates are considerably raised [7]. Acute psychiatric inpatient patients have a 100 times higher suicide rate than the global average during their inpatient stay or right after discharge, which is more than any comparable population in the world [8]. Recent Korean research reported that, between 2016 and 2018, the standardized mortality ratio for suicide within 30 days after psychiatric inpatient discharge was 66.8 compared with the general population [7]. In the United Kingdom, post-discharge suicides account for 17% of all patient suicides [9]. A study by Bickley et al., found that 55% of suicides occurred within the first week of discharge; the highest number of suicides occurred on the second day following discharge (*N* = 13, 24%). Nearly half (*N* = 24 of 49, 49%) had died before their first follow-up appointment [10]. Similarly Meehan et al., found that the most frequent post-discharge suicide happened in the first 2 weeks after leaving the hospital and the highest number occurred on the first day [11]. As a result of a meta-analysis that included 34 papers between 1945 and 2017 aimed at assessing the magnitude of suicide rates in the first week and the first month after psychiatric hospitalization, a total of 2,950 and 2,060 suicides per 100,000 person-years were estimated during the first week and month after discharge [7, 12]. To quantify the rates of suicide after discharge from psychiatric facilities, the team conducted a further meta-analysis of 100 articles published between 1946 and 2016. It has been observed that suicide rates remain high after discharge from psychiatric facilities for several years [13], but are particularly high in the first few months [11, 14] and weeks following discharge [11, 15].

Several studies have examined the potential risk factors associated with patients who experienced suicidal thoughts or committed suicide after discharge. Most studies indicate that suicide ideation and behavior are more common in men [13], except for one study, which found that more female patients died from suicide in the first week after discharge [16]. Additionally, studies indicate that people who are older than 40 [11], unmarried [16], unemployed [17], and living alone or having low levels of social support [17, 18] are at a high risk of becoming suicidal post-discharge. The most common mental health diagnoses associated with suicidal thoughts and actions are depression and schizophrenia [16, 17, 19]. Other clinical risk factors include non-adherence to treatment [16, 17, 19], a brief final admission [10, 11, 20, 21], and a history of/current self-harm [19, 22]. Furthermore, Chock et al. found that patients with a significantly higher number of outpatient, inpatient, and emergency department visits are at higher risk for developing suicidal ideation and committing suicide after discharge compared to the general population [23].

As psychiatric patients about to be discharged from hospitals represent an especially vulnerable population, it is crucial to evaluate suicide risk accurately at discharge and identify those individuals who are at greatest risk within the population of hospitalized psychiatric patients as the first step toward prevention. This study examined the prevalence of suicidal ideation and its associated risk factors among Alberta psychiatric patients nearing discharge.

## Methodology

### Study setting and design

The study was conducted in Alberta, Canada, which has an estimated population of 4.7 million [24] We recruited patients from Alberta hospitals about to be discharged in 7 days and invited them to complete baseline surveys as part of this epidemiological study. Discharge decisions are made at inpatient multidisciplinary team (MDT) meetings which are attended by the most responsible psychiatrists, nursing staff, social workers, psychologists, and occupational therapists. Decisions regarding discharge readiness are made by the MDT by consensus based on patients’ responses to treatment and positive changes in mental status. No randomization or interventions were used as the aim was to examine the prevalence and potential risk factors for suicidal ideation among patients who were about to be discharged from the hospital.

### Data collection and inclusion criteria

Face-to-face discussion were conducted from March 8, 2022 to November 5, 2023, to recruit participants across ten major sites in Edmonton, Calgary, and Grand Prairie in Alberta as part of a larger study [25]. Operational managers and clinical staff supported the research team by identifying patients about to be discharged in 7 days from a psychiatric unit. Eligible patients were provided with detailed study information. Written consent was obtained, and participants were invited to complete a self-administered questionnaire on a tablet device. The survey was designed on REDCap, an online survey platform [26]. Sociodemographic (e.g., age, sex, ethnicity, relationship status, etc.) and clinical information (e.g., diagnosis and levels of anxiety, depression, and well-being) were collected. Depending on participants’ discretion, researchers could either remain in the same room with participants or leave them alone. During the two-year recruitment, no participants indicated that they would like to be alone while filling out the questionnaire; instead, many of them indicated that they would like researchers to stay with them so they can receive prompt support when needed. All eligible participants were included in the analysis.

Inclusion and exclusion criteria: Participants must be diagnosed with at least one mental illness, were in the process of being discharged within 7 days from an inpatient psychiatric unit. They must be at least 18 years old and owned a mobile device with an active phone number. Participants also had to be capable of receiving texts, reading English texts, and providing written consent. This study excluded patients who planned to leave town during the six-month follow-up period because, as part of the main study [25], a peer support service intervention required patients to meet in person with peer support workers. Researchers obtained participant phone numbers and healthcare numbers for identification.

### Ethics statement

The study was approved by the Health Research Ethics Board at the University of Alberta (Ref # Pro00111459). Additional operational approval was also provided by the regional health authority. All participants provided written informed consent.

### Outcome measures

Patient Health Questionnaire-9 (PHQ-9).

The PHQ-9 is a self-report measure based on the 9 DSM-IV criteria for major depression. Item 9 of the PHQ-9 is used in research to assess the presence of suicidal ideation [27]. Therefore, item 9 is sometimes called the PHQ-9 suicide question because it determines the frequency of passive accidents caused by death or self-harm over the past two weeks [27–29]. Additionally, item 9 of PHQ-9 may be used as a predictor of suicidal death or risk even in the case of reduced depression severity [30]. A higher score on question 9 is more likely to predict suicide risk and mortality [31]. The PHQ-9’s ninth point, which is the key dependent variable, “Thoughts that you would be better off dead, or hurting yourself in the last two weeks,” was initially categorized into four categories (Not at all, Several days, More than half the days, and Nearly every day). This was broken down into two categories for analysis purposes: PHQ-9 ≥ 1 and PHQ-9 < 1 (thoughts that you would be better off dead or that you have hurt yourself in some way over the last two weeks, and having no thoughts of either). Several studies have used the PHQ-9 to detect patients at risk of suicide and have shown that suicidal ideation reported on the PHQ-9 was a robust predictor of suicide attempts and deaths regardless of age, and this increased risk persisted for two years [27, 30–34]. A study showed that the PHQ-9 suicide item had a specificity of 0.84 and sensitivity of 0.69, suggesting that the routine use of the PHQ-9 may be useful in primary care practice in that it may identify individuals at risk for suicide who would not otherwise have been identified [29]. Other clinical measures were assessed using validated scales for self-reported symptoms, including the Generalized Anxiety Disorder 7-item (GAD-7) scale [35] (for likely generalized anxiety disorder; GAD-7 ≥ 10) and the World Health Organization Well-Being Index (WHO-5) [36] (For poor emotional well-being: WHO-5 < 50).

### Statistical analysis

SPSS for Mac, version 25 (IBM et al., USA [37]) was used to analyze the data for this study. Age categories, 25 years or less, 26–40 years, and > 40 years were plotted against all independent variables. As part of univariate analysis, Chi-square was employed as an exploratory analysis to examine associations between sociodemographic/clinical variables and the categorical variable “Thoughts that you would be better off dead or hurting yourself” (Table 1). Variables with a statistically significant relationship with suicidal ideation (Bonferroni-corrected *p* ≤ 0.002) in the univariate analysis, as well as variables with non-significant associations (0.002 < *p* ≤ 0.1) but potential confounding effects on the outcome of interest, were included in the logistic regression analysis. This analysis was the planned analysis to examine the study’s main outcome. Variables were removed if they were strongly correlated with other variables included in the model (rs > 0.7).The decision to remove a variable that is highly correlated with another was based on the researchers’ assessment of the variable of most interest. The odds ratio (OR) and confidence intervals (CI) were used to assess the likelihood of developing suicidal ideas/thoughts (Table 2). The likelihood of developing suicidal ideas/thoughts was reported as an overall percentage. Results were presented in frequencies and percentages, with a critical significance level of *p* ≤ 0.05 defining statistical significance.

**Table 1:** Chi-squared test of association between demographic and clinical characteristics and likelihood of experiencing suicidal ideation in the preceding two weeks

Variables		Thoughts that you would be better off dead or of hurting yourself in some way in last two weeks *N* (%)	Have no thoughts that you would be better off dead or of hurting yourself in some way in last two weeks *N* (%)	Chi Square	*p*-value	Effect size
**Age**	≤ 25y	210 (59)	149 (41)	20.81	<0.001*	0.144
	26-40y	151 (43)	197 (57)			
	> 40y	129 (44)	167 (56)			
**Gender**	Male	195 (46)	231 (54)	7.66	0.022	0.087
	Female	275 (50)	274 (50)			
	Other	20 (71)	8 (29)			
**Ethnicity**	Caucasian	321 (51)	303 (49)	20.24	<0.001*	0.142
	Indigenous	57 (60)	38 (40)			
	Black people	33 (32)	70 (68)			
	Asian	50 (45)	61 (55)			
	Mixed/Other	29 (41)	41 (59)			
**Education Level**	Less than High School	22 (56)	17 (44)	1.34	0.72	0.037
	High School	252 (49)	263 (51)			
	Post-secondary Education	199 (48)	218 (52)			
	Prefer not to say	17 (53)	15 (47)			
**Relationship Status**	Single	292 (49)	299 (51)	6.88	0.14	0.083
	Separated or Divorced	45 (56)	35 (44)			
	Married/Partnered/Common-Law	131 (45)	160 (55)			
	Widowed	3 (30)	7 (70)			
	Prefer not to say	19 (61)	12 (39)			
**Employment Status**	Employed	169 (57)	129 (43)	16.61	0.002*	0.129
	Unemployed	243 (45)	292 (55)			
	Student	40 (52)	37 (48)			
	Retired	19 (33)	39 (67)			
	Other	19 (54)	16 (46)			
**Housing Status**	Own Home	88 (43)	117 (57)	7.36	0.061	0.086
	Rented Accommodation	170 (53)	154 (48)			
	Live with Family or Friends	195 (47)	216 (53)			
	“Couch surfing”/Shelter/Street/Other	37 (59)	26 (41)			
**Primary Mental Health Diagnosis**	Depression	168 (64)	94 (36)	79.70	<0.001*	0.282
	Bipolar Disorder	78 (38)	128 (62)			
	Anxiety Disorder	58 (43)	77 (57)			
	Schizophrenia	62 (39)	99 (61)			
	Personality Disorder	69 (77)	21 (23)			
	Substance Use Disorder	20 (39)	31 (61)			
	Other	35 (36)	63 (64)			
**GAD-7**	Unlikely Anxiety	204 (33)	422 (67)	177.16	<0.001*	0.422
	Likely Anxiety	282 (76)	88 (24)			
**WHO-5**	Good Wellbeing	153 (30)	366 (70)	161.56	<0.001*	0.401
	Poor Wellbeing	337 (50)	513 (50)			

*Significant *p* values

**Table 2:** Logistic regression predicting the likelihood of residents presenting suicidal ideation

		*P*-Value	Odd’s ratio	95% C.I.for EXP(B) Lower Upper	
**Age**	≤ 25y	0.001*			
	26-40y	< 0.001*	0.478	0.319	0.717
	> 40y	0.012*	0.539	0.332	0.874
**Gender**	Male	0.124			
	Female	0.045*	0.718	0.519	0.993
	Other	0.960	1.027	0.363	2.906
**Ethnicity**	Caucasian	0.040*			
	Indigenous	0.487	1.213	0.704	2.089
	Black people	0.005*	0.456	0.264	0.785
	Asian	0.441	0.820	0.495	1.359
	Mixed/Other	0.330	0.741	0.404	1.356
**Employment status**	Employed	0.033*			
	Unemployed	0.035*	2.062	1.051	4.048
	Student	0.550	1.211	0.647	2.270
	Retired	0.974	0.985	0.381	2.541
	Other	0.395	0.1556	0.562	4.306
**Housing status**	Own Home	0.199			
	Rented Accommodation	0.178	1.365	0.868	2.148
	Live with Family or Friends	0.346	1.273	0.771	2.104
	Coach surfing/Shelter/Street/Other	0.039*	2.159	1.038	4.489
**Primary Mental Health Diagnosis**	Depression	< 0.001*			
	Bipolar	< 0.001*	0.391	0.249	0.613
	Anxiety	< 0.001*	0.392	0.235	0.651
	Schizophrenia	< 0.001*	0.405	0.246	0.666
	Personality	0.110	1.695	0.887	3.239
	Substance use	0.037*	0.462	0.223	0.954
	Other	< 0.001*	0.345	0.193	0.619
**GAD-7 at baseline**	Likely anxiety	< 0.001*	4.556	3.263	6.361
**WHO-5 at baseline**	Poor wellbeing	< 0.001*	2.823	2.014	3.957
**Constant**		0.220	0.602		

C.I.: Confidence interval. S.E.: Standard error. Df: Degree of freedom. *: Significant predictor.

### Sample size calculation

In Alberta, there were 28,571 discharges from psychiatric units in 2018, with a 95% confidence interval and a ± 3% margin of error, so the sample size needed for prevalence of suicidal ideation (primary outcome measure) was 1,029 using an online script [38].

## Result

### Baseline data analysis

**Fig. 1:**
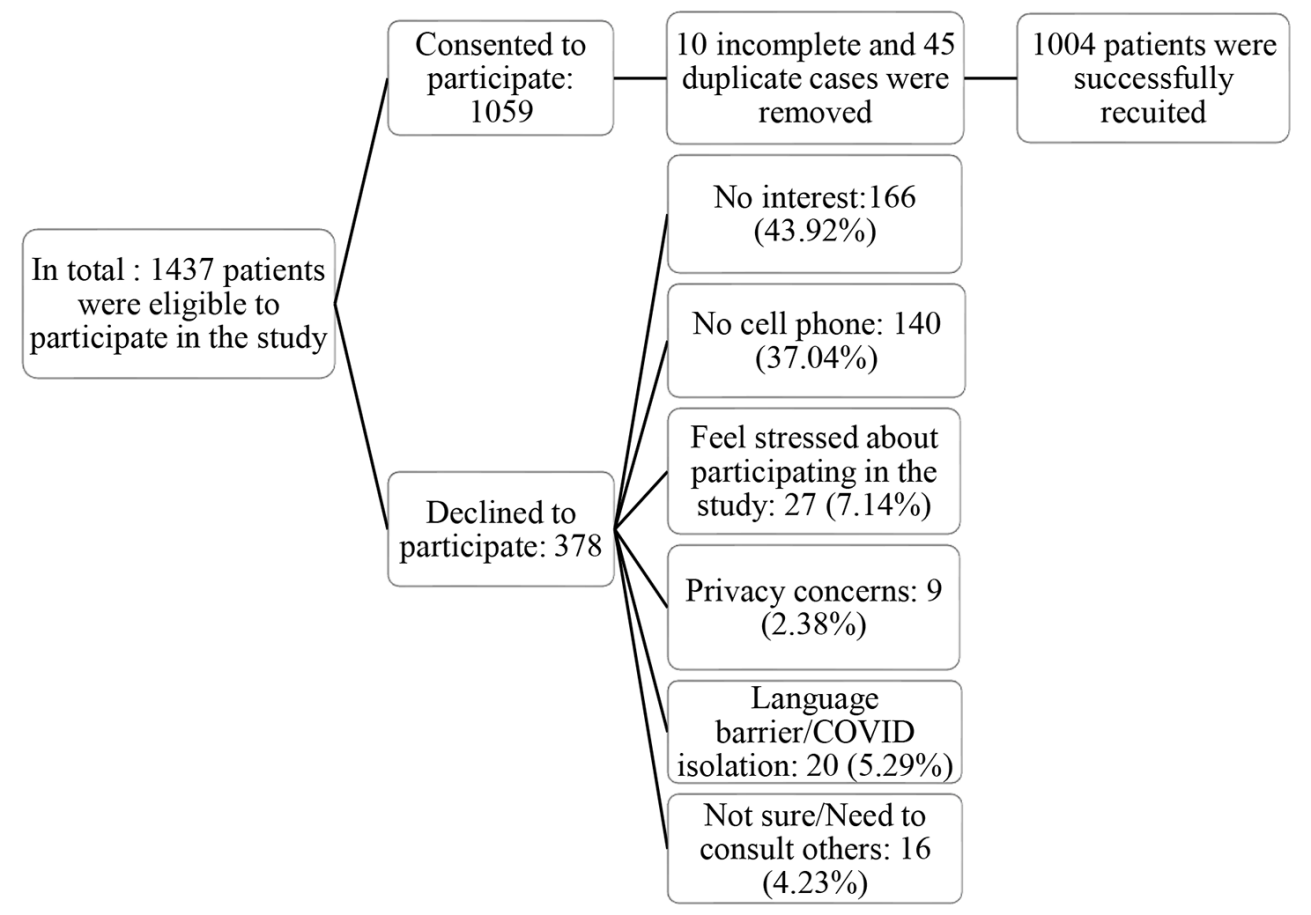
The flow chart of the recruitment process

Out of 1,059 responses, 1,004 responses remained after removing ten grossly incomplete cases and 45 duplicates (Fig. 1). Demographic and clinical data were collected from the entire sample of participants (*N* = 1,004), with age as a comparator variable (Table 3). The sample included 360 (35.8%) participants who were younger than 25 years old; 348 (34.7%) respondents were between 26 and 40 years old; 296 (29.5%) were older than 40 years old. A total of 426 (42.4%) respondents were male, 550 (54.8%) were female, and 28 (2.8%) were other genders. The majority of respondents, 625 (62.3%), were Caucasian; 536 (53.4%) were unemployed; 591 (58.9%) were single; 516 (51.4%) had a high school diploma; 412 (41.0%) lived with family or friends, and 262 (26.1%) were diagnosed with depression. In terms of the prevalence of clinical conditions, 370 (37.1%) of our respondents met the criteria for likely GAD, 566 (56.4%) met the criteria for likely MDD, and 484 (48.3%) met the criteria for poor well-being. It was reported by 490 participants (48.9%) that they had thought they would be better off dead or hurting themselves in some way in the past two weeks before being discharged from the hospital. Table 3 provides a more detailed description of the characteristics of the respondents.

**Table 3:** Distribution of socio-demographic and clinical characteristics among the study participants

Variables	Age			
	≤ 25 years (*N* = 426) *n* (%) = 42.4%	26–40 years (*N* = 550) *n* (%) = 54.8%	> 40 years (*N* = 28) *n* (%) = 2.8%	Total (*N* = 1,004)
**Gender**				
Male Female Other gender	141 (39) 202 (56) 17 (5)	156 (45) 181 (52) 11 (3)	129 (44) 167 (56) 0 (0)	426 (42) 550 (55) 28 (3)
**Ethnicity**				
Caucasian Indigenous Black people Asian Mixed/Other	191 (53) 39 (11) 52 (14) 55 (15) 23 (7)	221 (63) 28 (8) 37 (11) 34 (10) 28 (8)	213 (72) 28 (10) 14 (5) 22 (7) 19 (6)	625 (62) 95 (10) 103 (10) 111 (11) 70 (7)
**Education Level**				
Less than High School High School Diploma Post-secondary Education Prefer not to say	13 (4) 262 (72) 72 (20) 13 (4)	17 (5) 151 (43) 175 (50) 5 (2)	9 (3) 103 (35) 170 (57) 14 (5)	39 (4) 516 (51) 417 (42) 32 (3)
**Relationship Status**				
Single Separated or Divorced Married/Partnered/Common-Law Widowed Prefer not to say	273 (76) 0 (0) 65 (18) 0 (0) 22 (6)	216 (62) 23 (6) 103 (30) 3 (1) 3 (1)	102 (35) 57 (19) 124 (42) 7 (2) 6 (2)	591 (59) 80 (8) 292 (29) 10 (1) 31 (3)
**Employment Status**				
Employed Unemployed Student Retired Other	80 (22) 194 (54) 68 (19) 0 (0) 18 (5)	122 (35) 207 (60) 8 (2) 3 (1) 8 (2)	96 (32) 135 (46) 1 (0) 55 (19) 9 (3)	298 (30) 536 (53) 77 (8) 58 (6) 35 (3)
**Housing Status**				
Own Home Rented Accommodation Live with Family or Friend Couch surfing/Shelter/Street/Other	16 (4) 72 (20) 252 (70) 20 (6)	62 (18) 134 (38) 128 (37) 24 (7)	127 (43) 118 (40) 32 (11) 19 (6)	205 (20) 324 (33) 412 (41) 63 (6)
**Primary Mental Health Diagnosis**				
Depression Bipolar Disorder Anxiety Schizophrenia Personality Disorder Substance Use Disorder Other	90 (25) 62 (17) 52 (15) 57 (16) 58 (16) 12 (3) 29 (8)	77 (22) 84 (24) 38 (11) 70 (20) 30 (9) 18 (5) 31 (9)	95 (32) 60 (20) 45 (15) 34 (12) 3 (1) 21 (7) 38 (13)	262 (26) 206 (21) 135 (13) 161 (16) 91 (9) 51 (5) 98 (10)
**GAD-7**				
Unlikely Anxiety Likely Anxiety	215 (60) 143 (40)	215 (62) 132 (38)	196 (67) 95 (33)	626 (63) 370 (37)
**PHQ-9**				
Unlikely Depression Likely Depression	137 (38) 222 (62)	158 (45) 190 (55)	142 (48) 154 (52)	437 (44) 566 (56)
**WHO-5**				
Good Wellbeing Poor Wellbeing	175 (49) 184 (51)	198 (57) 150 (43)	146 (49) 150 (51)	519 (52) 484 (48)
**Thoughts That You Would Be Better off Dead or Hurting Yourself in Last Two Weeks**				
Absence of Suicidal Thoughts Presence of Suicidal Thoughts	149 (41) 210 (59)	197 (57) 151 (43)	167 (56) 129 (44)	513 (51) 490 (49)

### Univariate Analysis

Table 1 summarizes the results of the univariate analysis of the relationship between likely suicidal ideations and demographics and clinical experiences. Applying the Bonferroni corrected *p* value of 0.002, only six variables were significantly related to suicidal ideation. Younger respondents were more likely to present with suicidal ideation than those who were older. In comparison with females and males, other genders were more likely to experience suicidal ideation. People identifying as Indigenous are more likely to experience suicidal thoughts than other ethnicities. Further, people who were employed were more likely to exhibit suicidal ideation. In terms of mental health diagnoses, participants diagnosed with personality disorders were among the most likely to develop suicidal ideation. Additionally, suicidal ideation was more likely to occur in patients who were experiencing moderate to severe anxiety or poor well-being while about to be discharged. Though the association between gender and suicidal ideation was not statistically significant (*p* = 0.022), patients who self-identified as “other gender” had a trend to be more likely to report suicidal ideation compared to patients who identified as either male or female, thus making gender a potential confounding variable of interest. Similarly, housing status was not significantly associated with suicidal ideation. However, those living in less stable housing conditions (i.e., “Couch surfing”/Shelter/Street/Other) demonstrated a trend towards being more likely to have suicidal ideation than those who owned their own homes or lived with family or friends, thus making housing status a potential confounding variable of interest.

### Logistic regression analysis results

We entered the eight variables that were identified by the univariate analysis as either statistically significant or potentially confounding to predictors of suicidal ideation into the logistic regression model to predict the likely suicidal ideation. The complete model was statistically significant, *Χ*^2^ (23) = (385.88, *p* < 0.001), indicating that the model could distinguish between respondents who did or did not exhibit likely suicidal ideation at the time of inpatient discharge. The model accounted for 30.3% (Cox and Snell R^2^) and 40.3% (Nagelkerke R^2^) of the variance and correctly classified 74.6% of cases.

As shown in Table 2, age, ethnicity, employment status, primary mental health diagnoses, likely anxiety, and poor well-being at baseline could significantly predict participants’ potential suicidal ideation at the time of hospital discharge. There was an approximately two-fold increase in suicidal ideation among people younger than 25 years old at hospital discharge compared with people between 26 and 40 years old (OR = 0.48; 95% CI: 0.32-0.72) and those over 40 years old (OR = 0.54; 95% CI: 0.33-0.87). Black patients were over two time less likely to develop suicide ideation compared to Caucasians (OR = 0.46; 95% CI: 0.26 − 0.79). Unemployed individuals were twice as likely to report suicide ideations compared to those employed. Compared to primary mental health diagnosis of depression, the likelihood of presenting suicide ideation before discharge was approximately 2 to 3 times higher than for those diagnosed with a bipolar disorder (OR = 0.39; 95% CI: 0.25 = 0.61), anxiety disorder (OR = 0.39; 95% CI: 0.24 − 0.65), schizophrenia (OR = 0.41; 95% CI: 0.25 − 0.67), substance abuse disorder (OR = 0.46; 95% CI: 0.22 − 0.95) and other diagnoses (OR = 0.35; 95% CI: 0.19 − 0.62). Participants who met the criteria for likely GAD at hospital discharge were more than four times more likely to develop suicidal ideation than participants with at most mild anxiety (OR = 4.56; 95% CI: 5.26–6.36). In addition, those who met the criteria for poor well-being at discharge were almost four times more likely to experience suicidal ideation than those with good well-being (OR = 3.82; 95% CI: 2.01–3.96) while controlling for other factors. Similarly, although housing conditions did not contribute significantly to the model, people who were couch surfing or lived in shelters, on the street, or in other situations were more than two times more likely to present with suicidal ideation than those who owned their home (OR = 2.16; 95% CI: 1.04–4.49).

## Discussion

This study examined the prevalence and predictors of suicidal ideation among patients at discharge from psychiatric units across the province of Alberta. Approximately half of the study participants (48.9%) reported having considered death or harming themselves in some way within the two weeks prior to their discharge from psychiatric hospitals. This result found much higher rates of suicidal thinking than found in community samples, where the suicidal rate documented in the 2021 Health Reports in Canada was 4.8% [39]. Additionally, we found that the primary mental health diagnoses and sociodemographic factors, including age, ethnicity, and employment status significantly predicted pre-discharge suicidal ideation. Although we found no previous research regarding suicidal ideation among patients discharged from the psychiatric unit, our findings are in line with some other studies that measured suicidal thoughts and behaviour shortly after discharge [7, 8, 10–12].

Although research findings to date are inconsistent, age has been identified as a key factor significantly related to suicidal ideation with replication across multiple studies. In a study that aimed to investigate suicidal ideation among young adults in Canada during the COVID-19 pandemic, the authors found that suicidal ideation prevalence was the highest in younger age groups [40]. It is consistent with a United States study that reported that among respondents aged 18 to 24 years, the prevalence of serious suicide considerations during the past 30 days in 2020 was higher than among those aged 55 to 64 years [41]. The elderly have been reported to be less vulnerable to the psychological effects of stress in several publications [42–44]. Two theories have linked old age and mental health. With the “maturation theory,” older adults are believed to possess more mature coping mechanisms that protect them from stressors [45]. Due to this, elderly people tend to react less negatively to stressful events in their lives. Inoculation theory suggests that the result may be due to older individuals’ expertise and experience in developing coping skills in the face of challenges [45]. Contrary to our study, other research work, however, have demonstrated that middle-aged and older individuals are more likely to express suicidal thoughts and behaviors [46–49]. Physical illness, unemployment/financial problems, loneliness, cognitive impairment, or loved ones loss were among the most common causes [49]. A theory called “Resource Theory” supports this idea by stating that elderly individuals have more psychological problems and are unable to fully recover due to their lower socioeconomic status and limited functional ability [47, 48].

The results of our study revealed noteworthy findings regarding ethnicity. It was found that Caucasian patients were twice as likely as black patients to have suicidal ideation at discharge Several studies supported our finding and indicated that Caucasians were more likely than other ethnic groups to attempt suicide [50, 51], compared to Black [52] and Hispanic [53] identifying individuals. Furthermore, some [51], but not all [54], studies indicated that suicide attempt rates vary among different subgroups of Caucasians. In contrast, two national studies did not find a significant relationship between ethnicity and suicide ideations/attempts [55, 56]. Graham and Pinto explained the result by focusing on optimism and resilience, which may constitute a protective factor [57, 58]. Based on a study of the poor, controlling for sociodemographic factors, Low socioeconomic status blacks [RS1] were by far the most optimistic group and were nearly three times more optimistic than low socioeconomic status whites [58] Also, blacks and Hispanics report improved lives compared to their parents more often than whites. Despite ongoing disadvantages and discrimination, minorities are making gradual progress toward closing the gaps between them and whites in education, wages, and life expectancy [58]. Graham and Pinto also noted that resilience - defined as an individual’s capability of adapting successfully to stressful situations and maintaining psychological well-being despite adverse circumstances - may be higher among blacks and minorities due to their experience with adversity [58]. Additionally, community and religious factors may be involved; a cross-tabulation of data in Graham’s study indicated that blacks were the most likely of all racial groups to emphasize religion as significant in their lives [58]. On the contrary, several studies claimed suicidal-related problems were underestimated among black people [59, 60]. Warshauer et al. Indicated that Black people’s suicide rate was underestimated by 80% [60]. Some of the reasons may be lack of access to medical professionals, lack of information when investigating Black deaths, stigma associated with suicide within the Black community, etc [59].

In agreement with our findings, much of the existing literature indicates that keeping people employed is likely to reduce suicidal ideation and attempts [61–64]. Result of our study showed that, unemployed participants were two times more likely to experience suicidal ideation prior to hospital discharge compared with employed participants. According to literatures, several explanations exist [64]: stressful life events may impact an individual’s vulnerability due to unemployment; it may indirectly contribute to suicide by increasing the risk of factors that may precipitate suicide (for example, mental illness, financial hardship); or it may be a non-causal association as a result of confounding or selection by factors predicting both unemployment status and suicide risk [64]. Some studies have found the opposite results [61, 65]. A survey conducted by the U.S. Department of Health and Human Services found that approximately 11% of the 582 workers reported suicidal ideation, while 3% had moderate/severe suicidal ideation [65]. Job strain (a combination of low job control and high job demands) and long work hours (> 40 h per week) are associated with moderate to severe suicidal ideation in a working population, after controlling other factors [65]. Suicidal ideation was approximately four times more likely in those who reported job stress or long work hours [65]. Meanwhile, numerous studies report that suicidal ideation and attempts are highly prevalent among students, making them the most at-risk group, which makes future research essential to test the results [66, 67].

There is no surprise that individuals with pre-existing depression diagnoses were at the highest risk of exhibiting suicidal ideation upon discharge than people with other mental health diagnoses. People suffering from mental illness, particularly depression, are more likely to exhibit suicidal ideation [55, 68, 69]. As reported by the Substance Abuse and Mental Health Services Administration, nearly 30% of people who suffer from depression also experience thoughts of suicide [70]. In patients with untreated depression, the lifetime suicide risk is approximately 20% [71]. Among patients who have been treated for depression, the suicide risk is 141/100,000 [72]. Nearly two-thirds of people who commit suicide are believed to be depressed near the time of their death [72]. Psychiatrists and scholars have long recognized that depression can result in suicide ideation and suicidal crisis, and clinicians often consider these in their clinical assessment of depression. For any patient, this assumption is reasonable [70]. A person suffering from depression, insomnia, anhedonia, and hopelessness about the future may easily conclude that life is not worth living, particularly if there is no immediate solution to these problems [70]. In addition, the absence of joy and pleasure, a diminished ability to concentrate, unpleasant future expectations, as well as feelings of worthlessness and guilt, can also lead to a desire to die [70].

Apart from the primary mental health diagnosis, we also examined the level of anxiety and well-being of participants with self-administered GAD-7 and WHO-5 questionnaires. Suicidal ideation was much more likely to occur in people who had experienced likely anxiety and poor well-being conditions than those who did not meet these criteria. Understandably, that poor well-being can contribute to suicidal thoughts [73]. However, the relationship between anxiety disorders and suicidal ideation and behavior has been a subject of debate in the literature [74]. Several studies conducted across cross-sectional communities and in clinical settings have consistently found that anxiety disorders can predict an increase in suicidal ideation, suicidal behavior, attempts, and death [74, 75]. In other studies, anxiety was not associated with suicidal ideation or behavior or was inversely related [76, 77]. As anxiety disorders are highly comorbid with each other and tend to cluster together [74], it is essential to examine whether anxiety disorders, as a group of mental disorders, are associated with suicidal behavior after adjusting for other types of mental disorders (especially mood disorders and substance abuse disorders) [74].

Gender was not a significant predictor in our study, however, females tended to be less likely to have suicidal ideation than males. Although research indicated that suicide rates are 2 to 4 times higher for males than for females, suicide ideations have been found 3 to 9 times more prevalent in females [78–80]. This discrepancy is known as “the gender paradox” [81]. Annual data from the Australian Institute of Health and Welfare (2021) reported that suicidal ideation rates were 2.7% for women and 1.9% for men in the 12 months before the survey administration [82]. It is possible that this paradox is caused by the underreporting of ideation by males, and the differences in help-seeking behavior between genders due to a perceived availability of social support [83]. A central component of these explanations is the fact that there may be different links between distress and suicidal ideation for men and women as a result of differences in gender norms and expectations [83].

Likewise, although housing status was not a significant predictor in our study, individuals who reported living in places such as couch surfing, shelters, streets, or rented accommodations were found to be more likely to have suicidal ideation than those who own their own homes. It has been found that people become depressed and suicidal when they are exposed to repeated, uncontrollable environments that cause discomfort and suffering without an escape route [84]. Housing status is perceived as one of the main health determinants causally related to mental health. Those with less severe mental health challenges can experience aggravated symptoms if they lack a stable place to live [85]. Recent research suggests that stable housing reduces anxiety and suicidal thoughts and improves variables influencing mental health, such as depression and stress [86]. Suicidal thought is reasonable for patients awaiting discharge, when they do not know where they will live after discharge, or what they may expect to be a challenging environment and poor living conditions.

## Limitations

There are some limitations in the present study. For example, with a sample size of 1,004 rather than the 1,029 we had expected, our estimations of suicidal ideation prevalence had a margin of error of ± 3.09% at 95% confidence intervals rather than the ± 3% that we had anticipated. As well, self-reported measures were used in the study, including GAD-7, PHQ-9, and WHO-5 Well-being Index, which may cause biases and adversely affect the objectivity of the results. On top of that, the assessment of suicidal ideation was based on one question from the PHQ-9 rather than on an independent scale. On top of that, the assessment of suicidal ideation was based on one question from the PHQ-9 rather than on an independent scale. Furthermore, information about hospital services and patient care, such as the type of treatment, length of treatment, and admission mental state, was not assessed and may have had an effect on participants’ post-discharge mental health. It also needs to be recognized that as many as 50% of psychiatric inpatients do not fully disclose their suicidal thoughts [87], and it is possible that this is also the case for the current study. Even with the aforementioned limitations, this study is nonetheless valuable because it is a large sample of patients which further contributes to the relatively limited literature available in the mental health field concerning discharge and recovery in Canada and beyond.

## Conclusion

In this study, the prevalence and potential risk factors associated with suicidal ideations were assessed among Albertan patients who were about to be discharged from psychiatric units. In total, 490 participants (48.9%) reported considering death or harming themselves in some way within the past two weeks prior to filling out the survey. It was found that age, ethnicity, employment status, primary mental health diagnoses, likely anxiety, and poor well-being at baseline were significant predictors of participants’ potential suicidal ideation upon discharge from the hospital.

Clinicians should be aware of the increased risk of suicide in the immediate post-discharge period by people with suicidal ideation or thoughts at the time of discharge. This information must be incorporated into discharge and safety planning, where possible in collaboration with patients, their families, or caregivers, which includes an assessment of the patient’s social support system. Immediately following discharge, an integrated effort is required to support the patient in coping with the challenges of returning home. Considering that discontinuation of treatment is associated with an increased chance of suicidal behavior, it is important to address this issue in the immediate time following discharge.

## Data Availability

The datasets used and /or analysed during the current study are available from the corresponding author on reasonable request.
